# Supplementary material for: HIVprotI: an integrated web based platform for prediction and design of HIV proteins inhibitors
Source: J Cheminform. 2018 Mar 9;10:12. doi: 10.1186/s13321-018-0266-y (PMC5845081; doi:10.1186/s13321-018-0266-y)
Supplement: Supplementary file 2 — Additional file 2. Source code of HIVProtI web server. [file 13321_2018_266_MOESM2_ESM.zip › HIVprotI_Source-code/hivprotI/weka-3-6-6/doc/weka/gui/graphvisualizer/package-tree.html]

weka.gui.graphvisualizer Class Hierarchy


---


|  |  |  |
| --- | --- | --- |
| **Overview** \| **Package** \| Class \| **Tree** \| **Deprecated** \| **Index** \| **Help** \| **Weka's home** | |  |
| **PREV**   **NEXT** | **FRAMES**    **NO FRAMES**     **All Classes** |  |


---


## Hierarchy For Package weka.gui.graphvisualizer

**Package Hierarchies:**: All Packages

---

## Class Hierarchy

- java.lang.Object
  - weka.gui.graphvisualizer.**BIFParser** (implements weka.gui.graphvisualizer.GraphConstants)- java.awt.Component (implements java.awt.image.ImageObserver, java.awt.MenuContainer, java.io.Serializable)
      - java.awt.Container
        - javax.swing.JComponent (implements java.io.Serializable)
          - javax.swing.JPanel (implements javax.accessibility.Accessible)
            - weka.gui.graphvisualizer.**GraphVisualizer** (implements weka.gui.graphvisualizer.GraphConstants, weka.gui.graphvisualizer.LayoutCompleteEventListener)
      - weka.gui.graphvisualizer.**DotParser** (implements weka.gui.graphvisualizer.GraphConstants)- java.util.EventObject (implements java.io.Serializable)
          - weka.gui.graphvisualizer.**LayoutCompleteEvent**
          - weka.gui.graphvisualizer.**GraphEdge**- weka.gui.graphvisualizer.**GraphNode** (implements weka.gui.graphvisualizer.GraphConstants)- weka.gui.graphvisualizer.**HierarchicalBCEngine** (implements weka.gui.graphvisualizer.GraphConstants, weka.gui.graphvisualizer.LayoutEngine)- weka.gui.graphvisualizer.**Messages**- java.lang.Throwable (implements java.io.Serializable)
                    - java.lang.Exception
                      - weka.gui.graphvisualizer.**BIFFormatException**

## Interface Hierarchy

- weka.gui.graphvisualizer.**GraphConstants**- weka.gui.graphvisualizer.**LayoutCompleteEventListener**- weka.gui.graphvisualizer.**LayoutEngine**

---


|  |  |  |
| --- | --- | --- |
| **Overview** \| **Package** \| Class \| **Tree** \| **Deprecated** \| **Index** \| **Help** \| **Weka's home** | |  |
| **PREV**   **NEXT** | **FRAMES**    **NO FRAMES**     **All Classes** |  |


---
